# Cognitive Impairment in Adolescent Major Depressive Disorder With Nonsuicidal Self-Injury: Evidence Based on Multi-indicator ERPs

**DOI:** 10.3389/fnhum.2021.637407

**Published:** 2021-02-24

**Authors:** Yujiao Wen, Xuemin Zhang, Yifan Xu, Dan Qiao, Shanshan Guo, Ning Sun, Chunxia Yang, Min Han, Zhifen Liu

**Affiliations:** Department of Psychiatry, The First Hospital of Shanxi Medical University, Taiyuan, China

**Keywords:** cognitive impairment, adolescent, major depressive disorder, nonsuicidal self-injury behavior, event-related potential

## Abstract

The lifetime prevalence of major depressive disorder (MDD) in adolescents is reported to be as high as 20%; thus, MDD constitutes a significant social and public health burden. MDD is often associated with nonsuicidal self-injury (NSSI) behavior, but the contributing factors including cognitive function have not been investigated in detail. To this end, the present study evaluated cognitive impairment and psychosocial factors in associated with MDD with NSSI behavior. Eighteen and 21 drug-naïve patients with first-episode MDD with or without NSSI (NSSI+/– group) and 24 healthy control subjects (HC) were enrolled in the study. The Hamilton Anxiety Scale (HAMA), Hamilton Depression Scale (HAMD), Adolescent Self-injury Questionnaire, Beck Scale for Suicide Ideation–Chinese Version (BSI-CV), Shame Scale for Middle School Students, Sensation Seeking Scale (SSS) and Childhood Trauma Questionnaire (CTQ) were used to assess depression-related behaviors, and event-related potentials (ERPs) were recorded as a measure of cognitive function. The latency of the N1, N2, P3a, P3b, and P50 components of ERPs at the Cz electrode point; P50 amplitude and P50 inhibition (S1/S2) showed significant differences between the 3 groups. CTQ scores also differed across three groups, and the NSSI– and NSSI+ groups showed significant differences in scores on the Shame Scale for Middle School Students. Thus, cognitive function was impaired in adolescents with MDD with NSSI behavior, which was mainly manifested as memory decline, attention and executive function deficits, and low anti-interference ability. We also found that childhood abuse, lack of social support, and a sense of shame contributed to NSSI behavior. These findings provide insight into the risk factors for MDD with NSSI behavior, which can help mental health workers more effectively diagnose and treat these patients.

## Introduction

Major depressive disorder (MDD) is a common chronic mental disease characterized by persistent sadness, apathy, and anhedonia. MDD is associated with high rates of morbidity, recurrence, and suicide and has a low cure rate, and is often accompanied by cognitive impairment (Bayes and Parker, 2018). The prevalence of MDD is 4.4% worldwide and 4.2% in China (World Health Organization, 2017). Among adolescents in China, the rate of MDD is 15–20% and the lifetime prevalence may be as high as 20%, with a male-to-female ratio of 1:2 (Zheng et al., 2018).

Nonsuicidal self-injury (NSSI) behavior involves direct, intentional injury to one's body without suicidal intent, and is socially and culturally unacceptable (Ross and Heath, 2002). Common forms of NSSI include skin or wrist cutting, hair pulling, head hitting, biting, beating, scalding, acupuncture, pinching, etc. (Leong et al., 2014). NSSI behavior is listed as an independent clinical disorder in the Diagnostic and Statistical Manual of Mental Disorders, 5th Edition (DSM-V) (Andover, 2014; Zetterqvist, 2015). The incidence of NSSI behavior among adolescents is 10–20% (Zetterqvist et al., 2013; Célia et al., 2017). The co-occurrence of NSSI behavior with MDD in adolescents is mainly related to difficulties in interpersonal relationships, low self-esteem, childhood abuse, and lack of social support (Jiang et al., 2011; VanDerhei et al., 2014; Barreto Carvalho et al., 2017; Wang et al., 2017). One study found that shame and guilt were significant positive and negative predictors, respectively, of NSSI behavior (Xie et al., 2007).

Cognitive distortions and negative cognition contribute to adolescent suicide (Xie et al., 2017). Event-related potentials (ERPs) reflect brain activity and are a reliable indicator of cognitive function, and are thus used to diagnose diseases (Zhang et al., 2019). P1 latency was shown to be significantly delayed in patients with depression, suggesting a poor ability to attend to and discriminate between stimuli (Zhang et al., 2007; Liu W. et al., 2017; Liu Y. H. et al., 2017). Most studies have used ERPs to explore cognitive function in patients with depression but few have focused on adolescent NSSI behavior, although one study demonstrated that ΔFN [To objectively assess initial response to reward, they utilized the feedback negativity (FN) event-related potential, a well-established psychophysiological marker of reward responsiveness. ΔFN (i.e., FN to losses minus FN to gains)] is a psychophysiologic indicator of NSSI risk (Tsypes et al., 2018).

We speculated that cognitive deficits underlie NSSI behavior, and that adolescents with MDD with NSSI behavior show specific alterations in cognitive function. To test this hypothesis, we measured ERPs in adolescent patients with MDD and compared these findings to behavioral test scores from a battery of neuropsychological tests, with the aim of clarifying the features of and factors that contribute to MDD with NSSI behavior.

## Materials and Methods

### Participants

The Research Ethics Committee of Shanxi Medical University First Hospital approved the study protocol. The study included 63 subjects aged 10–22 years: 39 drug-naïve patients with first-episode MDD and 24 HC individuals. The drug-naive, first-episode MDD participants were recruited from the Department of Psychiatry, First Hospital of Shanxi Medical University, Taiyuan, China. The HC subjects were recruited from Taiyuan, China, using advertisement in the community. All participants were evaluated by two trained psychiatrists independently to determine the presence or absence of Axis I psychiatric diagnoses using the Structured Clinical Interview for Diagnostic and Statistical Manual of Mental Disorders, Five Edition (DSM-V) Axis I Disorders (SCID).

#### Inclusion and Exclusion Criteria for Patients With MDD

The inclusion criteria for MDD patients were as follows: (1) age between 10 and 22 years with no restrictions on gender; (2) met the DSM-V diagnostic criteria for MDD; (3) 24-item Hamilton Depression Scale (HAMD-24) score ≥20; (4) first-episode MDD with no previous use of antidepressant or other psychotropic medications; and (5) volunteered to participate in the study and signed the informed consent form. The exclusion criteria were as follows: (1) previous manic or hypomanic episodes; (2) any co-occurring mental disorder; (3) alcohol dependence or abuse; (4) hereditary and organic diseases; (5) intellectual disability; (6) personal or family history of epileptic seizures; (7) history of electroconvulsive therapy; (8) visual or hearing impairment; and (9) other severe physical disabilities or disorders.

#### Inclusion and Exclusion Criteria for HC Subjects

Inclusion criteria for HC subjects were as follows: (1) age 10–22 years; (2) no mental disorder found in the initial screening; (3) matched to the MDD patients in terms of sex and education level; and (4) participated voluntarily and signed the informed consent form. The exclusion criteria were as follows: (1) organic disease; (2) alcohol abuse within 30 days or alcohol or drug dependence within 6 months prior to the screening; (3) participation in other clinical trials in the previous 3 months; and (4) other conditions that disqualified the subject from the study, as determined by the investigators.

### Measures

Eligible participants were asked to provide sociodemographic information including name, gender, age, education years, occupation, ethnicity, residence, religious affiliations, etc. For correlations between clinically related variables and neural measures, we used the HAMD-24, Hamilton Anxiety Scale (HAMA) to assess the severity of depressive and anxiety symptom. Beck Scale for Suicide Ideation–Chinese Version (BSI-CV) was used to evaluate suicide ideation and attempts.

Behavior and severity of NSSI was assessed using the Youth Self-injury Questionnaire, a 18-items self-report scale that assesses NSSI behavior and severity; According to the assessment of the number of NSSI in the “past year,” it was divided into four grades: 0, 1, 2–4, 5 and above, and the score was 0–3. The assessment of the degree of physical injury was divided into five grades: no, mild, moderate, severe and extremely severe, and the score was 0–4. Eligible patients were categorized in the NSSI group (NSSI+) if they have self-injurious behavior. Patients were included in No self-inflicted injury group (NSSI–) if they don't have self-injurious behavior. The final subgroups included 18 patients categorized as NSSI+ and 21 as NSSI–.

Sensation Seeking Scale (SSS) and Shame Scale for Middle School Students were used to assess social support and shame; Childhood abuse was assessed using the Childhood Trauma Questionnaire (CTQ). The questionnaire consists of five subscales, namely emotional abuse, physical abuse, sexual abuse, emotional neglect, and physical neglect. Each subscale contains five items, and each item is rated on a five scale.

Cognitive function was evaluated by measuring ERPs (P300, N400, N170, and P50 were used to assess, respectively, executive function and memory, language function, face recognition ability and ability to selectively process stimuli).

### ERP Parameters

ERP data were collected using the 64-electrode NEMUS 2 system (EB Neuro, Florence, Italy). Recording electrodes were placed at the Fz, Cz, and Pz positions; the electrode at the Cz position was the standard and those at the Fz and Pz positions were references for waveform identification. Reference electrodes were placed on the mastoid processes (M1 and M2), and the ground electrode was placed in the middle of the parietal lobe.

#### P50 Detection

We measured the auditory ERP P50 component in response to 500-Hz, 60-dB short-range pure tones presented 32–64 times in pairs with superposition. The interval between the first and second stimuli (S1 and S2, respectively) was 0.5 s, with a paired stimulus interval of 10 s. The task had a total duration of 6 min. Electrode resistance was <5 kΩ; a bandpass filter of 0.1–300 Hz was applied; and the data were segmented into the time window from −200 to 800 ms.

#### P300 Detection

The task employed the classic oddball experimental paradigm. The stimulus sequence was composed of a target stimulus (T) and nontarget stimulus (NT) at a probability ratio of 0.2/0.8; T was randomly interspersed among NT, and the task consisted of 60 T and 240 NT. Subjects were required to press a key as soon as T appeared. The stimulus frequency was 0.5–1 time/s; stimulus interval was 1–3 s; and total task duration was 14 min. Electrode resistance was <5 kΩ; bandpass filtering was applied at 0.5–200 Hz; and the time window for data segmentation was −200 to 1,200 ms.

#### N400 Detection

The subjects were required to sit in a chair with their muscles relaxed and remain awake with eyes fixed on the screen. Three words were sequentially displayed on the screen, and subjects judged whether they could form a logical sentence (e.g., “Xiaoming,” “in the playground,” and “playing football”). Each word was presented for 100 ms and the time interval between presented words was 1,000 ms, giving the subjects 1,100 ms to respond. The total duration of the task was 3 min. Electrode resistance was <5 kΩ; the filter range was 0.53–60 Hz; and the time window for data segmentation was −200 to 1,000 ms.

#### N170 Detection

The procedure was similar to that used for N400 detection, except that subjects were presented with images instead of words and had to judge whether these were emotional or nonemotional. Each image was displayed for 300 ms; the time interval between images was 1,500 ms; and total task duration was 8 min. Electrode resistance was <5 kΩ, with bandpass filtering between 0.1 and 100 Hz and data segmented into the time window of −200 to 800 ms.

### Statistical Analysis

Data were analyzed using SPSS v22.0 (SPSS Inc., Chicago, IL, USA). The threshold of statistical significance was set as α = 0.05 for all analyses.

For general demographic data, categorical variables were evaluated with the χ^2^ test and continuous variables were evaluated with the *t*-test or by analysis of variance (ANOVA), which was used for HAMD-24, HAMA, CTQ, SSS, Youth Self-injury Questionnaire, BSI-CV, and Shame Scale for Middle School Students scores. ANOVA was also used to analyze ERP indicators, *post-hoc* analysis was then used to compare the ERP indicators between groups; the major components of ERPs were identified and their index values determined according to the internationally recognized maximum waveforms of the time analysis window.

Pearson's correlation analysis was performed to determine the relationship between the scores of Adolescen Self-injury Questionnaire and the scores of CTQ, SSS and shame scale in NSSI+ group (MDD with NSSI behavior). The results were considered significant if *P* < 0.05, corrected by Bonferroni test.

## Results

### Demographics, Clinical, and Psychosocial Characteristics of all Participants

The NSSI–, NSSI+ and HC groups showed significant differences in education years (*P* < 0.001); Covariance analysis showed that the influence of education years on the NSSI severity was not affected by grouping (*P* > 0.05) (Table 1; Supplementary Table 1).

**Table 1:** Demographic, clinical, and psychosocial characteristics of all participants (*N* = 63).

**Variable**	**NSSI+**	**NSSI–**	**HC**	***χ^2^/F/t***	***P***
Gender				4.179	0.128
Male	7	10	10		
Female	11	11	14		
Age, years	17.11 ± 2.54	18.91 ± 2.77	20.29 ± 0.46	7.965	0.092
Only child				0.04	0.000
Yes	10	11	13		
No	8	10	11		
Education, years	10.17 ± 2.99	11.76 ± 2.88	15.04 ± 0.86	23.649	0.000
Residence				0.948	0.635
City	12	11	13		
Rural	6	10	11		
HAMD-24	27.61 ± 1.01	25.29 ± 0.94	2.708 ± 1.04	417.893	0.000
HAMA	19.94 ± 4.41	17.57 ± 3.07	1.458 ± 0.72	280.824	0.000
Adolescent self-injury questionnaire	31.78 ± 1.55	0.00 ± 0.00	0.00 ± 0.00	531.304	0.000
BSI-CV–suicide ideation^†^	12.11 ± 0.54	0.00 ± 0.00	0.00 ± 0.00	630.180	0.000
BSI-CV–suicide risk^‡^	26.57 ± 4.16	0.00 ± 0.00	0.00 ± 0.00	232.526	0.000
Emotional abuse^a^	10.67 ± 0.75	9.38 ± 1.18	5.63 ± 0.47	10.098	0.000
Physical abuse^b^	8 ± 0.79	7.9 ± 0.82	5.29 ± 0.29	6.048	0.004
Sexual abuse^c^	6.17 ± 0.65	5.67 ± 0.22	5 ± 0	2.894	0.063
Emotional neglect^d^	16.1 ± 1.21	15.08 ± 1.38	12.54 ± 0.62	3.323	0.043
Physical neglect^e^	10.72 ± 0.92	10 ± 0.64	6.33 ± 0.19	16.079	0.000
CTQ total score	49.22 ± 2.5	49.05 ± 2.64	13.13 ± 1.54	16.568	0.000
SSS total score	21.61 ± 0.35	24.67 ± 0.77	32.54 ± 1.57	0.418	0.660

*All subjects were students of Han ethnicity, not married, with no religious affiliation*.

*Data represent number, mean ± standard deviation*.

*BSI-CV, Beck Scale for Suicide Ideation–Chinese Version; HAMA, Hamilton Anxiety Scale; HAMD, Hamilton Depression Scale; CTQ, Childhood Trauma Questionnaire; SSS, Sensation Seeking Scale; HC, healthy control; NSSI, nonsuicidal self-injury; NSSI+, MDD with nonsuicidal self-injury; NSSI-, MDD with no self-inflicted injury*.

† *Score on the suicide ideation subscale of the BSI-CV*.

‡ *Score on the suicide risk subscale of the BSI-CV*.

a−e *Scores on the emotional abuse (a), physical abuse (b), sexual abuse (c), emotional neglect (d), and physical neglect (e) subscales of the CTQ*.

The three groups showed significant differences in HAMD-24, HAMA, Adolescent Self-injury Questionnaire, BSI-CV, emotional and physical abuse, emotional and physical neglect subscales of the CTQ scale scores (*P* < 0.05). Covariance analysis showed that the main effect between grouping and HAMA, HAMD, CTQ, SSS, and BSI-CV was not significant (*P* > 0.05).

There were no significant differences between the three groups in terms of age, gender, only-child status, residence and the SSS total score (*P* > 0.05). There were statistically significant differences in Shame Scale scores between the NSSI- and NSSI+ groups (*P* < 0.001).

### Group Differences in Cognitive Function

The results of the ERP analysis showed that compared to HC subjects, the latency of the N1, N2, P3a, P3b, and P50 components was significantly prolonged in the NSSI– and NSSI+ groups; additionally, the amplitude of P50 was decreased, and inhibition of P50 (S1/S2) was increased (*P* < 0.05) (Table 2). *Post-hoc* analysis showed that, there were no statistically significant differences between NSSI+ group and NSSI– group for the ERP components (*P* > 0.05); Compared to the HC group, P300 latency was longer in the NSSI– group and the NSSI+ group (*P* < 0.05). On the other hand, N400 latency were shorter, respectively, in the HC group than in the NSSI- group (*P* < 0.05). There were no other statistically significant differences between groups for the other ERP components (Table 3).

**Table 2:** ANOVA analysis of ERP results among the NSSI+, NSSI–, and HC groups.

**ERP component**		**NSSI+**	**NSSI–**	**HC**	***F***	***P***
Latency, ms	N1	131 ± 4.41	126 ± 4.78	115 ± 3.33	4.369	0.017
	P2	216 ± 3.28	215 ± 3.15	207 ± 2.58	2.894	0.063
	N2	250 ± 4.56	246 ± 4.29	230 ± 3.56	6.915	0.002
	P3a	343 ± 5.31	330 ± 3.99	313 ± 2.41	13.814	0.000
	P3b	373 ± 4.52	364 ± 5.03	343 ± 3.19	21.159	0.000
	L1	57.2 ± 2.9	55.3 ± 2.3	50.9 ± 1.74	4.718	0.043
	L2	56.7 ± 3.7	54.5 ± 3.2	49.7 ± 1.32	5.726	0.037
	N400	430 ± 4.67	429 ± 4.47	417 ± 3.74	2.564	0.085
	N170	192 ± 5.6	185 ± 5.03	177 ± 2.8	1.526	0.226
Amplitude, μV	N1	−2.5 ± 0.79	−1.9 ± 3.02	−1.5 ± 0.59	1.165	0.319
	P2	3.49 ± 0.87	3.51 ± 1.4	5.51 ± 1.71	0.969	0.385
	N2	2.01 ± 1.25	2.03 ± 0.7	2.54 ± 1.63	0.326	0.723
	P3a	9.8 ± 1.62	10.7 ± 2.5	12.7 ± 2.02	1.366	0.263
	P3b	10.8 ± 1.8	11.2 ± 0.7	13.4 ± 2.1	0.791	0.458
	S1	0.31 ± 0.21	0.52 ± 0.37	−0.49 ± 0.4	4.73	0.730
	S2	0.32 ± 0.26	0.45 ± 0.21	−0.37 ± 0.2	2.965	0.046
	N400	6.26 ± 0.99	5.47 ± 1.67	4.81 ± 2.29	0.509	0.603
	N170	2.86 ± 1.34	2.35 ± 1.01	1.34 ± 0.63	1.800	0.174
S1/S2		0.54 ± 0.13	0.55 ± 0.3	0.48 ± 0.21	5.826	0.008

*Data represent mean ± standard deviation*.

*ERP, event-related potential; HC, healthy control; NSSI, nonsuicidal self-injur; NSSI+, MDD with nonsuicidal self-injury; NSSI-, MDD with no self-inflicted injury*.

**Table 3:** *Post-hoc* analysis of ERP latency among the NSSI+, NSSI– and HC groups.

**ERP component**		**NSSI+ vs. NSSI–**	**NSSI+ vs. HC**	**NSSI- vs. HC**
Latency, ms	N1	−4.913(1.000)	−16.681(0.02)	−11.768(0.04)
	P2	−1.103(1.000)	−9.056(0.112)	−7.952(0.167)
	N2	−3.563(1.000)	−19.778(0.004)	−16.214(0.016)
	P3a	0.635(1.000)	−23.597(0.000)	−24.232(0.000)
	P3b	1.063(1.000)	−31.681(0.000)	−32.744(0.000)
	L1	1.161(0.705)	−1.297(0.039)	−2.458(0.048)
	L2	1.213(0.649)	−1.843(0.041)	−2.159(0.036)
	N400	0.317(1.000)	−11.278(0.205)	−11.595(0.153)
	N170	−74.476(0.431)	−77.708(0.350)	−3.232(1.000)
Amplitude, μV	N1	0.838(1.000)	−1.786(0.405)	0.948(1.000)
	P2	−2.225(0.632)	−2.021(0.723)	0.204(1.000)
	N2	−1.285(1.000)	−0.508(1.000)	0.778(1.000)
	P3a	−2.841(0.543)	−3.137(0.386)	−0.296(1.000)
	P3b	−2.577(1.000)	−2.389(1.000)	0.188(1.000)
	S1	−0.054(1.000)	−0.805(0.937)	−0.751(0.976)
	S2	0.251(1.000)	−0.591(0.746)	−0.649(0.859)
	N400	−2.281(0.965)	−1.492(1.000)	0.789(1.000)
	N170	0.052(1.000)	−2.447(0.372)	−2.499(0.304)
S1/S2		−1.384(1.000)	2.984(1.000)	3.142(1.000)

*Data represent mean difference (P)*.

*ERP, event-related potential; HC, healthy control; NSSI, nonsuicidal self-injury; NSSI+, MDD with nonsuicidal self-injury; NSSI–, MDD with no self-inflicted injury*.

### Correlation Between Psychosocial Factors and NSSI Severity

Pearson correlation was used to analyze the correlation between the NSSI severity and childhood abuse, social support and shame in NSSI+ group, and the results showed that NSSI severity was positively correlated with childhood abuse (*r* = 0.667, *P* < 0.01) and sense of shame (*r* = 0.776, *P* < 0.01), and negatively correlated with social support (*r* = −0.464, *P* < 0.01).

## Discussion

The present study explored cognitive impairment and psychosocial factors in first-episode untreated MDD patients with NSSI behavior. To the best of our knowledge, this study investigated for the first time the differences in cognitive function and psychosocial factors between patients diagnosed with major depression (with and without NSSI) and healthy controls. We found that the latencies on N1, N2, P3a, P3b, and P50 were significantly prolonged and the amplitudes on P50 were significantly decreased in NSSI+ group. The cognitive function of adolescents with self-injury behavior in MDD is impaired, which is mainly manifested as memory loss, dysfunction of attention and execution, and low ability of anti-interference. In addition, we also found that childhood abuse, lack of social support and sense of shame are all causes of self-injury.

### Cognitive Function to MDD With NSSI Behavior in Adolescents

As the main means and index to detect cognitive function, ERP has important clinical significance in the study of cognitive impairment in patients with depression. Patients with MDD have cognitive impairment to a certain extent, which is mainly related to the dysfunction of frontal lobe (executive function) and temporal lobe (memory) (Hansenne et al., 1996). Based on our findings, compared with NSSI- group and HC group, the latency of N1, N2, P3a, and P3b in NSSI+ group was significantly prolonged and the amplitude decreased. It is speculated that neuronal excitability and cognitive processing speed decreased in NSSI+ group, and there was some impairment of executive function and memory ability.

In this study, we found that the latency and amplitude of P50 in NSSI+ group were worse than those in NSSI– group, and the inhibition of P50 in P50 group was worse than that in NSSI– group. The P50 component of ERP reflects the selective processing of significant external stimuli in the brain. P50 inhibition-measured by S2/S1 ratio-is an indicator of screening or gating intensity (Wang et al., 2011). The weaker the gating function of MDD patients is, the lower the selective processing ability to important external stimuli is. Combined with previous studies (Wang et al., 2012), we found that there are some defects in the screening ability of unrelated stimuli in adolescent depression patients with NSSI behavior.

The N400 is used to test language processing ability; the N400 latency reflects the speed of semantic processing in the brain, while the amplitude reflects the speed at which words are processed in context. A smaller N400 amplitude indicates a higher speed (Kutas and Federmeier, 2011; Zhang et al., 2012). In this group of MDD patients with self-injury behavior, the latency of N400 was longer and the amplitude was higher, which is consistent with previous studies (Liang and Zhou, 2014). Compared with NSSI– group, NSSI+ group has more obvious language barrier, which is mainly manifested in slower language processing speed.

N170, ERP faces specific components, is a negative detection of the occipito-temporal region after 130–190 milliseconds of a face, and reflects the structure of the coding phase faced by the brain processing and early detection of face information (i.e., distinguishing face from non-face) (Itier and Taylor, 2004). The results showed that compared with NSSI- group, the latency of N170 in NSSI+ group was longer, the amplitude was lower, and the speed of face image recognition was slower, so we speculated that the face recognition ability of adolescent depression patients with NSSI behavior decreased.

### Psychosocial Factors Contributing to MDD With NSSI Behavior in Adolescents

#### Childhood Abuse Contributing to MDD With NSSI Behavior in Adolescents

Childhood abuse refers to various forms of physical or mental abuse, sexual abuse, neglect, commercial or other forms of exploitation that cause actual or potential harm to the health, survival, development and dignity of the child, subject to appropriate responsibilities and abilities (Yao et al., 2010). Severe or moderate abuse; emotional abuse; unwanted sexual contact; repeated contact sexual assault/non-contact sexual assault is one of the main risk factors for self-injury behavior (Yu et al., 2013). Our study found that the scores of emotional neglect, physical neglect, emotional abuse and physical abuse in the NSSI+ group were higher than those in the NSSI– group. Patients in the NSSI+ group often reported various kinds of abuse and neglect in childhood. Some literature also showed that emotional and physical abuse and neglect experienced in childhood were risk factors for self-injury behavior (Brodsky and Stanley, 2008; Fergusson et al., 2011). Therefore, based on our findings, we speculate that the more abuse and neglect experienced in childhood, the greater the probability of self-injury when they grow up.

Childhood abuse, as a negative experience, will affect the normal function of children's brain neurotransmitters and hormones, including the development of brain regions related to coping problems and emotional control. It will have a series of adverse effects on children's physical and mental health and the development of cognitive function, thus increasing the risk of adolescents' risky behaviors (Gilbert et al., 2015). Factors that affect NSSI behavior include all aspects, although we can't directly determine the childhood abuse and the inevitable cause-and-effect relationship between NSSI behavior (Su et al., 2015), but it exists as a kind of risk factors, should remind us to strengthen the neglect and abuse of children, to give children a certain support and unconditional love, prevent the happening of the risk behavior.

#### Social Support Contributing to MDD With NSSI Behavior in Adolescents

It is also worth noting that the study showed that the social support score of MDD patients (with or without NSSI) was lower than that of HC subjects, and the social support score of the NSSI+ group was also lower than that of the NSSI– group. Patients in the NSSI+ group often report less family support, their ideas are not understood by others, and there are few companions, so when they encounter stress or setbacks, they cannot or even will not seek help. At this time, self-injury has become an effective way for them to ease their emotions and relieve stress. NSSI behavior is not uncommon in adolescent students, and is particularly common in middle school students between the ages of 13 and 17. Adequate social support can significantly reduce the risk of mental health problems such as NSSI and suicide (Duggan et al., 2015). Conversely, a lack of social support and childhood abuse is linked to NSSI behavior in later life (Liu W. et al., 2017; Liu Y. H. et al., 2017).

Adolescent students are more sensitive, they have extremely unstable emotions, unpredictable and difficult behavior: storms and stress, they are still in an important period of physical and mental development, in this period, we cannot continue to use “storm and stress” to misinterpret adolescent problems. The research on the social development of teenagers mostly focuses on the changes of family and peer roles. In the interaction with their peers, teenagers gradually determine the social factors of their own identity in the process of development, and then determine what kind of person they become (Gao, 2013). During this period, parents' company, friends' communication and teachers' care all become powerful and effective sources of social support for them.

#### Shame Contributing to MDD With NSSI Behavior in Adolescents

In addition, we also observed the relationship between NSSI behavior and shame. In this study, self-injury was used as a way of self-punishment to study the relationship between NSSI behavior and shame. The results showed that the shame score of NSSI+ group was higher than that of NSSI– group and HC group. Guilt, shame and strong disgust increased before NSSI and decreased after NSSI. According to the results of the study, we found that shame is an important factor affecting self-harm behavior, with the increase of shame, the degree of self-harm will become more and more serious. This result has also been confirmed by many studies (Linehan, 1993; Tanaka et al., 2015).

It has been suggested that NSSI is the expression of anger toward oneself, and self-directed anger and self-deprecation are features of individuals who engage in NSSI (Tanaka et al., 2015; Wang et al., 2019). There are usually negative emotions before self-injury, leading to depression and self-hatred. Individuals take different behaviors to alleviate these emotions and achieve self-coordination and self-balance, including self-injury. In a study on the influencing factors of NSSI behavior among adolescents, it was found that 70% of teenagers reported “I don't like myself” and 63% chose to say “I'm mad at myself” (Laye et al., 2005). Therefore, self-punishment is one of the most common causes of self-injury behavior (Linehan, 1993). In real life, teenagers will have negative emotional experience when they encounter negative life events, and they lack effective ways to deal with emotions, so they choose self-injury to alleviate their negative emotions.

### Limitations

This study had certain limitations. Firstly, the sample size was small, and although we found evidence of cognitive impairment in adolescents with MDD with NSSI behavior, this needs to be validated in a larger cohort. Compared with healthy adolescents, MDD with NSSI behavior in adolescents have obvious cognitive impairment, but compared with adolescent depressive patients without NSSI behavior, there is no significant difference in cognitive function between the two groups. This may be due to the small sample size (18 patients categorized as NSSI+ and 21 as NSSI–), resulting in no significant difference between the two groups. Future studies should include more samples to verify. Secondly, this was a cross-sectional study and there was no long-term follow-up; in the future it would be useful to investigate whether interventions such as psychological counseling, drug treatment, or physical therapy can alter cognitive function in patients with MDD with comorbid NSSI behavior.

## Conclusion

Compared to adolescent patients with MDD with no self-inflicted injury, those with NSSI behaviors had significantly impaired cognitive function, which was mainly manifested as memory loss, inattention, reduced executive function, and poor resource utilization. Additionally, compared to HC subjects, adolescent patients with MDD with NSSI behavior had poor information screening and anti-interference abilities as well as deficits in language processing and face recognition and processing. The main psychosocial factors associated with NSSI in adolescents with MDD were childhood abuse, lack of social support, and a sense of shame. The results of this study highlight the risk factors for MDD comorbid with NSSI behavior, which can help mental health workers more effectively diagnose and treat these patients.

## Data Availability Statement

The raw data supporting the conclusions of this article will be made available by the authors, without undue reservation.

## Ethics Statement

The studies involving human participants were reviewed and approved by Ethics Committee of First Hospital of Shanxi Medical University. Written informed consent to participate in this study was provided by the participants' legal guardian/next of kin. Written informed consent was obtained from the individual(s), and minor(s)' legal guardian/next of kin, for the publication of any potentially identifiable images or data included in this article.

## Author Contributions

YW, YX, and DQ contributed to study design and were involved in data acquisition, analysis, and interpretation. XZ contributed to data acquisition. SG, NS, CY, MH, and ZL contributed to study design and data interpretation. All authors participated in the drafting or critical review of the article, gave final approval of the version to be published, and agree to be accountable for all aspects of the work.

## Conflict of Interest

The authors declare that the research was conducted in the absence of any commercial or financial relationships that could be construed as a potential conflict of interest.
