# Supplementary material for: Cognitive Impairment in Adolescent Major Depressive Disorder With Nonsuicidal Self-Injury: Evidence Based on Multi-indicator ERPs
Source: Front Hum Neurosci. 2021 Feb 24;15:637407. doi: 10.3389/fnhum.2021.637407 (PMC7943920; doi:10.3389/fnhum.2021.637407)
Supplement: Supplementary file 1 [file Table_1.docx]

# Supplementary materials

**Table S1.** Covariance analysis of education years among the NSSI+, NSSI- and HC groups

| Source | Type III Sum of Squares | df | Mean Square | F | P |
| --- | --- | --- | --- | --- | --- |
| Corrected Model | 13069.211 | 5 | 2613.842 | 230.137 | 0.000 |
| Intercept | 103.500 | 1 | 103.500 | 9.113 | 0.004 |
| Group | 994.612 | 2 | 497.306 | 43.786 | 0.000 |
| Age | 7.855 | 1 | 7.855 | 0.692 | 0.409 |
| Group * Age | 46.729 | 2 | 23.364 | 2.057 | 0.137 |
| Error | 647.392 | 57 | 11.358 |  |  |

Abbreviations: HC, healthy control; NSSI, nonsuicidal self-injury; NSSI+, MDD with nonsuicidal self-injury; NSSI-, MDD with no self-inflicted injury.
